# Sweet syndrome and acute pulmonary coccidioidomycosis in West Texas

**DOI:** 10.1016/j.jdcr.2024.03.001

**Published:** 2024-03-08

**Authors:** Maria Batchinsky, Corley Pruneda, Ethan Matthew, Michelle Tarbox

**Affiliations:** Department of Dermatology, Texas Tech University Health Sciences Center School of Medicine, Lubbock, Texas

**Keywords:** pulmonary coccidioidomycosis, Sweet syndrome

## Introduction

Sweet syndrome, or acute febrile neutrophilic dermatosis, is defined by acute painful, nonpruritic, erythematous nodules with histopathologic evidence of a neutrophilic infiltrate. Sweet syndrome may present concurrently with underlying infection, hematologic malignancy, or vaccination.^1^ Literature review yields sparse reports of Sweet syndrome in the setting of pulmonary coccidioidomycosis, particularly in Arizona and after travel to Mexico.^2^, ^3^ This case report highlights Sweet syndrome in conjunction with pulmonary coccidioidomycosis in a Texas patient.

## Case report

A 51-year-old man presented to an urgent care facility for temperatures up to 38.8 °C and a rash. Incidentally, a right sided lung nodule was found on chest x-ray with preliminary suspicion for malignancy due to distant smoking history. Follow-up with oncology was scheduled, and the patient was discharged from the facility with oral amoxicillin.

**Fig 1:**
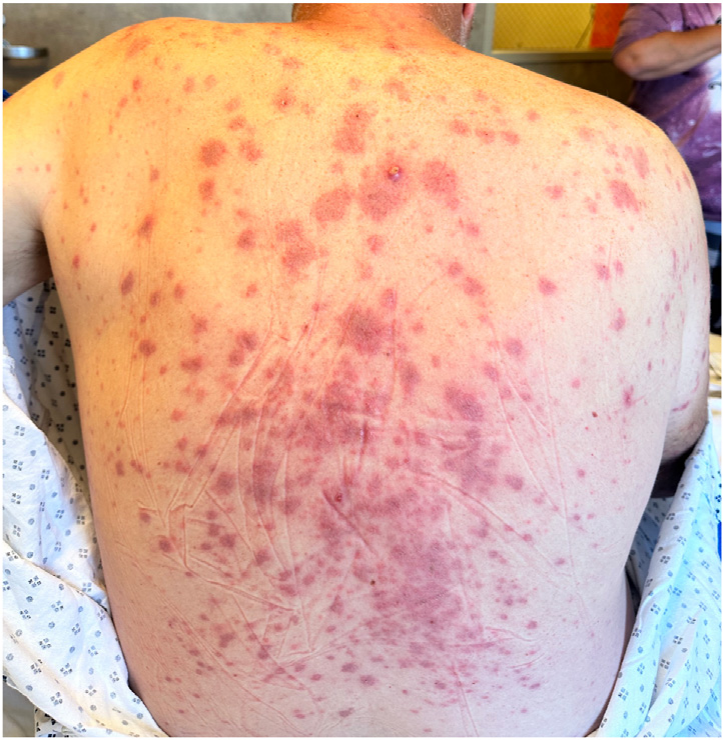
Cutaneous involvement of the back by Sweet syndrome presenting as multiple, red and violaceous papules and plaques.

Three days after this visit, the patient presented to the emergency department for continued rash, malaise, fatigue, and severe arthralgias of the bilateral ankles with inability to bear weight. He stated that these symptoms began 1.5 weeks before admission. Multiple 5- to 20-mm erythematous to violaceous papules and plaques were present on the knees, trunk, upper extremities, chest, and face but spared the mucous membranes, palms, and soles (Fig 1).

Although initially tender, with time the lesions became nontender and mildly pruritic. Lymphadenopathy of the left posterior cervical chain was noted, and review of systems was negative for ocular symptoms.

On admission the patient had elevated inflammatory markers (erythrocyte sedimentation rate, 78 mm/hr; C-reactive protein, 19.2 mg/L; white blood cell, 10.82 ×10^9^/L; with segmented neutrophils of 78.6% and procalcitonin of 0.18 ng/mL). Initially, infection was suspected along with potential underlying malignancy. Further history from the patient, who worked outdoors in West Texas, revealed initial symptoms of cervical lymphadenopathy and 24-lb weight loss began after disassembling an old shed 4 months before admission. One month before admission, the patient received 40 mg intramuscular triamcinolone injection and a 6-day methylprednisolone taper starting with 24 mg on day 1 and ending with 4 mg on day 6 for seasonal rhinitis. Because of unknown etiology of his presentation and possible infectious exposure, rare fungal infection was suspected, and titers were drawn. Immunologic workup was negative for baseline immunodeficiency or autoimmune disease with negative markers, including antinuclear antibody. Dermatology was consulted for the rash and 2 (4 mm) punch biopsies of the left thigh and mid upper portion of the back were submitted for hematoxylin-eosin staining, bacterial, and fungal cultures.

**Fig 2:**
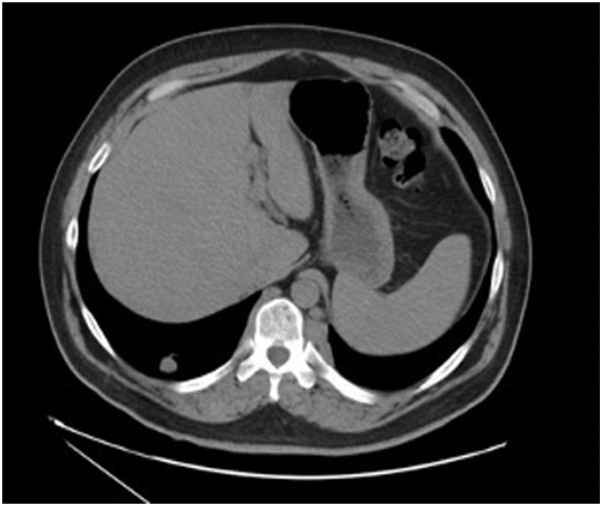
Computed tomography scan of right lower lobe nodule of the lung.

The patient underwent computed tomography imaging of the chest (Fig 2) illustrating a 1.5 cm spiculated right lower lobe lung nodule consistent with neoplastic process versus atypical infection. Additional sub-6 mm right lower lobe pulmonary nodules were noted, and computed tomography–guided tissue biopsy of the peripheral lung nodule was performed.

Because of significant improvement of rash during the inpatient stay and stable overall outlook, the patient was discharged with pending biopsy and fungal titer results. He was given 0.1% triamcinolone ointment at discharge for pruritus secondary to residual rash.

**Fig 3:**
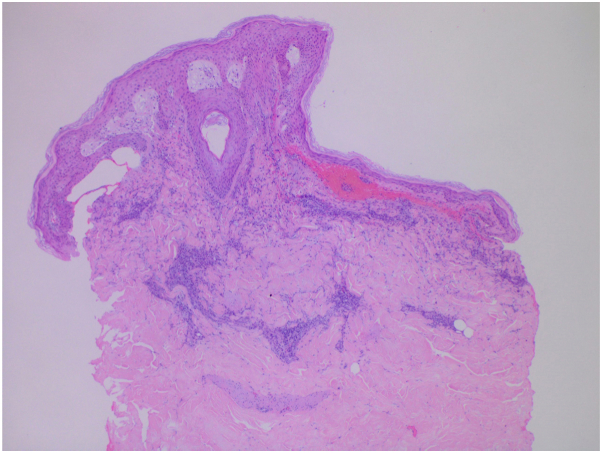
Significant papillary dermal edema overlying a perivascular and interstitial mixed inflammatory infiltrate with a predominance of neutrophils. (Original magnification: ×5.)

**Fig 4:**
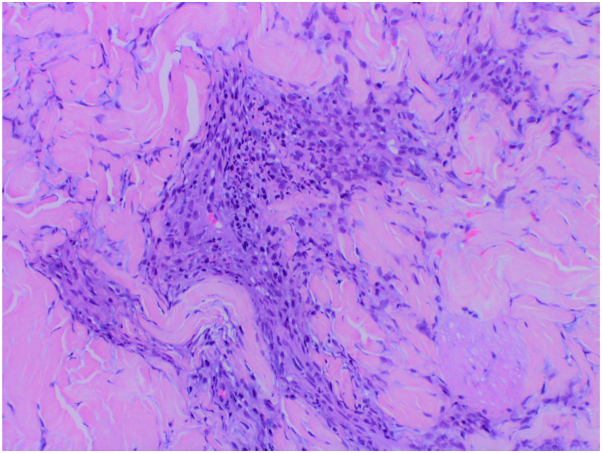
Perivascular neutrophils without leukocytoclastic vasculitis. (Original magnification: ×20.)

Several days after discharge, lung biopsy confirmed a lack of malignant cells and presence of large multinucleated giant cells with granulomatous formation indicating fungal infection. Coccidioides antibody complement fixation of 1:16 and IgG and IgM serologies were positive and laboratory markers were negative for other fungal etiology. Hematoxylin-eosin staining revealed significant papillary dermal edema overlying a perivascular and interstitial mixed inflammatory infiltrate with a predominance of neutrophils without evidence of leukocytoclastic vasculitis, consistent with a diagnosis of Sweet syndrome (Figs 3 and 4). Direct immunofluorescence studies were negative and tissue culture grew normal flora. Fungal culture of the initial biopsy remained negative for growth at 4 weeks.

The patient was prescribed 800 mg fluconazole daily for 12 weeks for treatment of pulmonary coccidioidomycosis and was instructed to follow-up in 2 weeks with the internal medicine team. At that time, he was tolerating fluconazole well with decreasing lung mass and resolved rash. The patient has yet to follow-up with dermatology; however, recurrence and resolution of the rash was documented by the infectious disease specialist.

## Discussion

The nature of abrupt onset of the rash, abnormal laboratory results, documented and reported temperature of 38.8 °C, and neutrophil-predominant inflammatory infiltrate on histology confirmed the diagnosis of Sweet syndrome. Thus, meeting 2 major and 3 of 4 minor diagnostic criteria, with confirmatory fungal titers, the diagnosis of pulmonary coccidioidomycosis was made. Because of Sweet syndrome’s rarity in relation to this underlying fungal infection, diagnostic workup should be thorough and involve dermatology colleagues for biopsy and diagnostic certainty.

Previously, DiCaudio et al described 2 cases of Sweet syndrome with presence of pulmonary nodules in association with coccidioidomycosis.^2^ Histological evaluation of cutaneous lesions included “mild edema of the subepidermal region and dense dermal inflammatory infiltrate”.^2^ In our case, histological findings were similar (Figs 3 and 4) and pathognomonic for Sweet syndrome. In the aforementioned cases, patients showed improvement of pulmonary symptoms within 5 weeks of treatment with antifungal agents of fluconazole and itraconazole, or amphotericin B in disseminated infection.^4^^,^^5^ Although Sweet syndrome responds clinically to systemic corticosteroids, in the setting of coccidioidomycosis Sweet syndrome may resolve without treatment in weeks to months.^4^^,^^6^ In the authors’ opinion, addressing the underlying fungal infection holds priority and systemic corticosteroids should be used with caution.

In this case, exposure to coccidiomycosis likely occurred months before admission and corticosteroid use may have delayed onset of Sweet syndrome in the setting of worsening coccidioidomycosis infection. Notably, his rash began to improve during the inpatient stay without topical or systemic corticosteroids, and complete resolution of the rash at 2 week follow-up visit was noted before later recurrence. Recurrence of Sweet syndrome may happen in one-third of patients; however, in cases associated with pulmonary coccidioidomycosis, such data are unreported.^7^ Systemic steroids are a consideration once treatment for coccidioidomycosis is completed.

In conclusion, Sweet syndrome is a rare cutaneous manifestation that has yet to be reported in Texas in a patient with confirmed pulmonary coccidioidomycosis. The thorough review of history played a vital role in the workup, whereas dermatology consult and skin biopsy proved essential in the diagnosis and treatment of this patient.

## Conflicts of interest

Disclosed individually by authors upon submission.
